# A case report of schizoaffective disorder with ritualistic behaviors and catatonic stupor: successful treatment by risperidone and modified electroconvulsive therapy

**DOI:** 10.1186/s12888-018-1655-5

**Published:** 2018-03-13

**Authors:** Yuanhan Bai, Xi Yang, Zhiqiang Zeng, Haichen Yang

**Affiliations:** Department of Affective Disorder, Shenzhen Kangning Hospital, Cuizhu Road, Luohu District, Shenzhen, 518020 China

**Keywords:** Schizoaffective disorder, Ritualistic behaviors, Catatonic stupor, Risperidone, Modified electroconvulsive therapy

## Abstract

**Background:**

Ritualistic behaviors are common in obsessive compulsive disorder (OCD), while catatonic stupor occasionally occurs in psychotic or mood disorders. Schizoaffective disorder is a specific mental disorder involving both psychotic and affective symptoms. The syndrome usually represents a specific diagnosis, as in the case of the 10th edition of the International Classification of Diseases (ICD-10) or the 5th edition of the Diagnostic and Statistical Manual of Mental Disorders (DSM-5). However, symptom-based diagnosis can result in misdiagnosis and hinder effective treatment. Few cases of ritualistic behaviors and catatonic stupor associated with schizoaffective disorder have been reported. Risperidone and modified electroconvulsive therapy (MECT) were effective in our case.

**Case presentation:**

A 35-year-old man with schizoaffective disorder-depression was admitted to the hospital because of ritualistic behaviors, depression, and distrust. At the time of admission, prominent ritualistic behaviors and depression misled us to make the diagnosis of OCD. Sertraline add-on treatment exacerbated the psychotic symptoms, such as pressure of thoughts and delusion of control. In the presence of obvious psychotic symptoms and depression, schizoaffective disorder-depression was diagnosed according to ICD-10. Meanwhile, the patient unfortunately developed catatonic stupor and respiratory infection, which was identified by respiratory symptoms, blood tests, and a chest X-ray. To treat psychotic symptoms, catatonic stupor, and respiratory infection, risperidone, MECT, and ceftriaxone were administered. As a result, we successfully cured the patient with the abovementioned treatment strategies. Eventually, the patient was diagnosed with schizoaffective disorder-depression with ritualistic behaviors and catatonia. Risperidone and MECT therapies were dramatically effective.

**Conclusion:**

Making a differential diagnosis of mental disorders is a key step in treating disease. Sertraline was not recommended for treating schizoaffective disorder-depression according to our case because it could exacerbate positive symptoms. Controversy remains about whether antipsychotics should be administered for catatonic stupor. However, more case studies will be needed. Risperidone with MECT was beneficial for the patient in our case.

## Background

Diagnosis is the first step toward correctly curing disease. Unlike internal or surgical diseases, mental disorders are largely symptom-based diagnoses [1, 2]. In the process of interviewing, syndromes are always associated with certain diagnoses according to the ICD-10 or DSM-5. Repetitive behaviors or ritualistic behaviors may be linked with OCD [3]. Immobility, mutism, negativism, and peculiar motor behavior represent catatonic stupor [1, 2], which is a psychotic diagnosis because approximately 10–15% patients with catatonic stupor meet the criteria for schizophrenia [4]. Typical ritualistic behaviors and catatonic stupor may represent OCD and psychotic disorders, respectively. However, these patients are not “textbook” cases, which means that complex and complicated symptoms may lead to misdiagnosis. Schizoaffective disorder is a specific mental disorder involving both psychotic and affective symptoms [5]. It is classified as “schizophrenia, schizotypal, and delusional disorders” by ICD-10 [1] and “schizophrenia spectrum and other psychotic disorders” by DSM-5 [2]. The complex symptomatology of schizoaffective disorder makes it highly likely that patients will be misdiagnosed.

Sertraline is a selective serotonin reuptake inhibitor [6] that is used to treat depression and OCD [7]. Previous studies have summarized the effective use of antidepressants in schizoaffective disorder [8]. However, the risk of exacerbation of positive symptoms of antidepressants should be considered [9–11]. A review of the treatments for catatonia has shown that MECT is effective, while antipsychotics remain controversial [12]. On the other hand, Huang et al. published other papers suggesting that the Lorazepam-Diazepam protocol can rapidly and safely relieve catatonia in schizophrenia, mood disorder, and organic lesions [13–15].

In this paper, we present the case of a patient who was initially suspected of having OCD but who actually suffered from schizoaffective disorder-depression with ritualistic behaviors and catatonic stupor. Sertraline exacerbated the psychotic symptoms. The ritualistic behaviors that were actually secondary to psychotic symptoms may have prevented us from making an accurate diagnosis. Finally, risperidone and MECT were effective strategies for this patient.

## Case presentation

The patient was a 35-year-old male. He was apparently normal before the age of 27 years without any medical problems. He was intellectually normal and worked as a security guard. He was never married and had no children, often living with his older sister. He had been smoking for approximately 10 years or more, denying alcohol or other psychoactive substance abuse. He is the third child, with one older brother and one older sister. His father often became drunk and violent, going outside for no reason, and committed suicide many years ago. There were no detailed records for his father because he failed to see a doctor.

In 2009, the patient gradually became depressive; showed diminished pleasure, insomnia, and fatigue; and was unwilling to talk to others. Meanwhile, he developed delusions of persecution and reference, which made him believe that someone had insulted him and planned to kill him without evidence. Later, he came to the outpatient clinic of our hospital and was prescribed paroxetine 20 mg/d and sulpiride 0.2 g/d. He took these drugs irregularly, with minimal improvement in depressive symptoms and delusions. In August 2013, the patient was sent to the hospital for schizoaffective disorder-depression. During the following month, with quetiapine 600 mg/d and lithium carbonate sustained-release tablet 0.6 g/d, his depressive and positive symptoms improved. Taking these drugs, he almost enjoyed normal life and work. Unfortunately, he discontinued these medications in May 2017. Again, the patient gradually developed fear of the sound of water, a lack of any pleasure and negative ideas, and claimed that he could not trust anyone. Furthermore, the patient performed ritualistic behaviors, such as walking with a specific order. Once again, he was sent to our hospital by his older sister. At the time of admission, the patient presented depressive symptoms as well as ritualistic behaviors and distrust.

Upon admission, liver and kidney function, routine blood test, computed tomography (CT) of the head, and electrocardiograph (ECG) were normal. Depressive symptoms, delusions, and ritualistic behaviors were found upon psychiatric interview. By day 9 in the hospital, we followed outpatient therapeutic strategies with quetiapine 100 mg/d and lithium carbonate sustained-release tablet 0.6 g/d, observing that the depressive symptoms and distrust had moderately improved. However, the ritualistic behaviors gradually worsened. Before waking up, he would swing his arms up and down four times, did sit-ups four or five times, and sat at the edge of the bed for a few minutes, all of which took him approximately 8 min. These disturbed or interrupted behaviors made the patient anxious. Because of the predominant ritualistic behaviors, OCD was suspected first. Here, we wanted to reduce these behaviors by adding sertraline at a dose of 50 mg/d and titrating it to 100 mg/d.

Then, the symptoms further worsened, and the patient developed agitation, pressure of thoughts, and delusion of control. He felt that his ritualistic behaviors gradually became out of control, realizing that “unknown thoughts” and “a black shadow” affected his mind. Meanwhile, he felt sad, and there was nothing that brought him pleasure. Considering the clinical picture and depressive and psychotic symptoms with equal importance, the diagnosis of schizoaffective disorder-depression was eventually made according to the ICD-10. Sertraline 100 mg/d was immediately ceased. We planned to change the ineffective quetiapine to risperidone, which was more effective on positive symptoms according to our own clinical experiences when the patient developed catatonia. Mutism, posturing, nonverbal communication, hyper-myotonia of the limbs, and saliva collected in the mouth were observed. Redness and swelling of the pharynx and hyperthermia (38 °C) were present. Routine blood tests showed that the white blood cell (WBC) count was 12.85 × 10^9^/L (normal range 3.5–9.5 × 10^9^/L), and the neutrophil granulocyte (NEUT) count was 10.6 × 10^9^/L (normal range 1.8–6.3 × 10^9^/L). Chest computed tomography indicated a high-density streak like a shadow in the lower lobe of the left lung, which was clear at admission. After the case discussion, we decided to initiate risperidone at a dose of 1 mg/d and gradually titrate it to 4 mg/d to control the positive symptoms. A twice-daily intravenous injection of ceftriaxone 1 g in 250 mL 0.9% physiological saline was administered to treat the respiratory infection. Meanwhile, MECT was added three times a week to ameliorate the catatonia. MECT was administered with the SPECTRUM-5000Q device used in the bilateral mode. The treatment parameters included frequency (30 Hz), stimulus duration (2.5 s), electric charge (120 Mc), energy (21.1 J), and constant current (800 mA). Vital signs were stable; specifically, body temperature was not over 37.2 °C before starting MECT in this patient. Before and during the MECT, anesthesia was induced with etomidate 10 mg and muscle relaxation with succinylcholine 50 mg, while arterial oxygen saturation, heart rate, and electrocardiogram were continuously monitored. Each time, the patient experienced adequate generalized seizures measured with an electroencephalogram. The patient was ventilated with 100% oxygen until the resumption of spontaneous respiration.

On hospital day 23, inflammation of the pharynx disappeared, and normal WBC and NEUT counts suggested that the respiratory infection had been clinically cured. Ceftriaxone was ceased when we found negative blood bacterial culture results. We continued risperidone and MECT (a total of nine times) treatments. The patient gradually began to talk with doctors and other patients, joining some activities in the ward. The pressure of thoughts and delusion of control almost disappeared. Furthermore, it took him less and less time to perform the ritualistic behaviors. On hospital day 31, we stopped MECT and added lithium carbonate sustained-release tablets 0.6 g/d. Oral risperidone 4 mg/d was introduced, in which the blood concentration was 8.7 μg/L (normal range 2–60 μg/L). No obvious side effects were observed. Finally, we titrated the lithium carbonate sustained-release tablets to 0.9 g/d and maintained risperidone 4 mg/d. The patient remained normopyretic, and his psychotic and depressive symptoms were stable. We told the patient to review and check the blood lithium carbonate concentration 1 week later in the outpatient setting.

For the publication of this case report, written informed consent was obtained from the patient and his older sister.

## Discussion

Here, we describe a case of schizoaffective disorder-depression with ritualistic behaviors and catatonia. After admission, we suspected a diagnosis of OCD because of the dominance of ritualistic behaviors and depression, as the patient reported that the ritualistic behaviors must be performed or else he would feel sad. The ritualistic behaviors proved to be secondary to delusion of control. The patient’s insomnia, fatigue, and unwillingness to talk to others were not explained by the ritualistic behaviors. Ultimately, we made a diagnosis of schizoaffective disorder-depression. The symptom-based diagnostic criteria [1, 2] as well as the multivariate symptoms made diagnosis and treatment difficult. Our case suggests that time, patience, and detailed observation are essential factors for making clinical decisions.

Selective Serotonin Reuptake Inhibitors (SSRIs) are beneficial for depression and OCD [6], among which sertraline is an effective therapeutic strategy [7]. In early studies and guidelines, psychiatrists were concerned about the risk of exacerbating psychosis when prescribing antidepressants to schizophrenic patients [9–11]. Rebecca Schennach et al. identified the exacerbation of positive symptoms in patients with antidepressant augmentation compared with patients without any antidepressants during the course of the study, and patients with antidepressant add-on treatment suffered from more severe psychopathological symptoms and greater psychosocial impairments at discharge [16]. However, a recent review does not support these points, as no studies found that add-on antidepressants worsened positive symptoms [17]. Although many studies have described the use of antidepressants in schizophrenia with depression, controversies remain about whether to administer antidepressants for schizophrenia spectrum disorders. We suggest that therapeutic strategies for schizoaffective disorder-depression might not include additional antidepressants, for sertraline may have exacerbated positive symptoms in our case.

Catatonia is a neuropsychiatric syndrome with psychomotor inhibition that occurs in approximately 8% of patients admitted for mental disorders, such as schizophrenia or mood disorders [18]. Schizophrenia and other psychotic spectrum disorders are more commonly presented as catatonia than mood disorders [19]. Catatonic stupor is a psychiatric emergency due to a broad range of complications [20, 21]. Neuroleptic malignant syndrome (NMS) typically presents with fever, muscle rigidity, and altered mental status [22] and should be differentiated from catatonic stupor complicated by respiratory infection. In this case, the clear consciousness of the patient with psychomotor inhibition, redness and swelling of the pharynx, fever without muscle rigidity, increased WBC and NEUT counts, and a high-density streak of shadowing in the lower lobe of left lung in the chest X-ray at admission suggested a status of catatonic stupor complicated by respiratory infection. Therefore, our case describes schizoaffective disorder-depression with catatonic stupor complicated by respiratory infection. Controlling the infection and improving the catatonic stupor were important in treating this patient. Three treatment strategies were employed in this case. Supportive measures included high-level nursing care, intravenous fluids, and gastrointestinal support to reduce the risk of bedsores and deep vein thrombosis caused by immobility and to improve poor nutrition and dehydration. Antibiotic treatment was considered due to the redness and swelling of the pharynx, hyperthermia, increased WBC and NEUT counts, and abnormal chest X-ray. These symptoms successfully responded to the administration of 7 days of ceftriaxone. MECT was an effective strategy for improving catatonic stupor [23, 24]. Although benzodiazepines proved to rapidly and effectively relieve catatonia, we did not prescribe these kinds of medications for the patient considering his poor nutrition, respiratory infection, and risk of respiratory inhibition. However, the role of antipsychotics in the treatment of catatonia is controversial. Several authors have suggested that antipsychotics may exacerbate the catatonic state and increase the risk of NMS [25, 26]. Studies have found that second-generation antipsychotics (SGAs) have weak γ-aminobutyric acid (GABA)-agonist activity and 5-hydroxytryptamine_2_ (5-HT_2_)-antagonism that could stimulate dopamine release in the prefrontal cortex and thus alleviate catatonic symptoms [20]. Several articles have suggested a beneficial effect of risperidone [27, 28]. A case report identified MECT together with olanzapine, which resulted in improvement of catatonic stupor [29]. Given the results of the abovementioned studies, risperidone was cautiously administered at a low dose (2 mg/d). Once catatonic stupor improved and MECT came to a stop, risperidone would be titrated from 2 to 4 mg/d to target residual psychotic symptoms, such as the pressure of thoughts and delusion of control. Furthermore, lithium carbonate is also an effective strategy for patients with schizoaffective disorder, as it can reduce the rate of re-hospitalization [30].

## Conclusion

Here, we describe a case of schizoaffective disorder-depression with secondary ritualistic behaviors complicated by catatonic stupor, which induces respiratory infection. Sertraline might not be recommended in patients with schizoaffective disorder to improve depression, which could have exacerbated the positive symptoms in our case. Supportive measures have an important role in the treatment of catatonic stupor. Once again, MECT has been shown to improve catatonic stupor. Although there is controversy over whether antipsychotics should be administered in the catatonic status, we considered risperidone to be beneficial in our case. Further studies should focus on the effectiveness and safety of antipsychotics associated with MECT in patients with catatonia.

## Abbreviations

5-HT_2_: 5-hydroxytryptamine_2_
CT: Computed tomography
DSM-5: 5th edition of Diagnostic and Statistical Manual of Mental Disorders
ECG: Electrocardiograph
GABA: γ-aminobutyric acid
ICD-10: 10th edition of International Classification of Diseases
MECT: Modified electroconvulsive therapy
NEUT: Neutrophil granulocyte
NMS: Neuroleptic malignant syndrome
OCD: Obsessive compulsive disorder
SGAs: Second-generation antipsychotics
SSRIs: Selective Serotonin Reuptake Inhibitors
WBC: White blood cell
